# Notch1 Deficiency Induces Tumor Cell Accumulation Inside the Bronchiolar Lumen and Increases TAZ Expression in an Autochthonous *Kras* ^LSL-G12V^ Driven Lung Cancer Mouse Model

**DOI:** 10.3389/pore.2021.596522

**Published:** 2021-04-16

**Authors:** Lydia Meder, Alexandra Florin, Luka Ozretić, Marieke Nill, Mirjam Koker, Sonja Meemboor, Freddy Radtke, Linda Diehl, Roland T. Ullrich, Margarete Odenthal, Reinhard Büttner, Lukas C. Heukamp

**Affiliations:** ^1^ Department I of Internal Medicine, University Hospital Cologne, Cologne, Germany; ^2^ Center for Molecular Medicine Cologne, University of Cologne, Cologne, Germany; ^3^ Institute for Pathology, University Hospital Cologne, Cologne, Germany; ^4^ Department of Cellular Pathology, Royal Free Hospital, London, United Kingdom; ^5^ École Polytechnique Fédérale de Lausanne, Swiss Institute for Experimental Cancer Research Lausanne, Switzerland; ^6^ Institute of Experimental Immunology and Hepatology, University Medical Center Hamburg-Eppendorf, Hamburg, Germany; ^7^ Institute for Hematopathology Hamburg, Hamburg, Germany; ^8^ Lungen Netzwerk NOWEL, Oldenburg, Germany

**Keywords:** mouse model, lung cancer, KRAS, NOTCH1, TAZ

## Abstract

**Purpose:** Abrogation of Notch signaling, which is pivotal for lung development and pulmonary epithelial cell fate decisions was shown to be involved in the aggressiveness and the differentiation of lung carcinomas. Additionally, the transcription factors YAP and TAZ which are involved in the Hippo pathway, were recently shown to be tightly linked with Notch signaling and to regulate the cell fate in epidermal stem cells. Thus, we aim to elucidate the effects of conditional Notch1 deficiency on carcinogenesis and TAZ expression in lung cancer.

**Methods:** We investigated the effect of conditional Cre-recombinase mediated *Notch1* knock-out on lung cancer cells *in vivo* using an autochthonous mouse model of lung adenocarcinomas driven by *Kras* ^LSL-G12V^ and comprehensive immunohistochemical analysis. In addition, we analyzed clinical samples and human lung cancer cell lines for TAZ expression and supported our findings by publicly available data from The Cancer Genome Atlas (TCGA).

**Results:** In mice, we found induction of papillary adenocarcinomas and protrusions of tumor cells from the bronchiolar lining upon Notch1 deficiency. Moreover, the mutated *Kras* driven lung tumors with deleted *Notch1* showed increased TAZ expression and focal nuclear translocation which was frequently observed in human pulmonary adenocarcinomas and squamous cell carcinomas of the lung, but not in small cell lung carcinomas. In addition, we used data from TCGA to show that putative inactivating *NOTCH1* mutations co-occur with *KRAS* mutations and genomic amplifications in lung adenocarcinomas.

**Conclusion:** Our *in vivo* study provides evidence that Notch1 deficiency in mutated *Kras* driven lung carcinomas contributes to lung carcinogenesis in a subgroup of patients by increasing TAZ expression who might benefit from TAZ signaling blockade.

## Background

Lung cancer is the leading entity in cancer-related mortality in men and women worldwide [1]. Approximately 85% of lung cancer cases belong in the group of non-small cell lung carcinomas (NSCLCs), comprising adenocarcinomas (ADCs), squamous cell carcinomas (SCCs) and large cell carcinomas [2]. Around 15% of lung cancer cases account for small cell lung carcinomas (SCLCs) [3]. About 4–9% of NSCLCs occurs as a mixed adeno-squamous carcinoma [4] and this mixed lung cancer subgroup is associated with a worse prognosis leading to significantly shortened survival after resection compared to carcinoma entities with unilinear differentiation [5].

ADCs are derived from alveolar type II, Clara cells and bronchioalveolar precursors located in the alveolar periphery and the bronchiolar epithelium [6, 7]. SCCs arise from basal cells residing below the epithelia of the upper airways expressing specific lineage markers as p63, cytokeratin (CK) 5 and 6 and thrombomodulin [4]. Frequent oncogenic drivers of ADCs are mutated *KRAS* and *EGFR*, which are less frequent in SCCs [2].

Inactivating mutations in Notch genes were identified in SCC of both, skin and lung [8]. The Notch pathway consists of four different membrane-spanning receptors (Notch1-4), which transduce canonical signals after binding their ligands (Jagged1 and 2, Delta 1, 3 and 4) on neighboring cells. Upon ligand binding, these receptors are cleaved at two proteolytic sites, which liberates the functionally active Notch intracellular domain (NICD) into the cytoplasm. NICD translocates to the nucleus and builds a DNA-binding complex together with its co-activator Mastermind-like (MAML) to regulate target gene expression. Thereby, Notch signaling fulfills pivotal functions in lung development and in epidermal cell differentiation [9].

Importantly, abrogation of Notch signaling by means of the mechano-activation of transcriptional coactivator with PDZ-binding motif (TAZ), encoded by the *WWTR1* gene, was recently shown to alter epidermal stem cell fate decisions [10]. TAZ is an effector of the Hippo signaling pathway and has a functional role in organ development. Moreover, TAZ is implicated as an oncogene in NSCLC [11], and TAZ expression is associated with worse prognosis and is additionally an independent prognostic factor for poor survival in patients with resected NSCLC [12].

In our study, we aimed to investigate the effects of conditional Notch1 deficiency on carcinogenesis and TAZ expression using an autochthonous *Kras* ^LSL-G12V^ driven lung cancer mouse model [13].

## Methods

### Animal Experiments

This study was carried out in accordance to the recommendations of the Federation of European Laboratory Animal Science Association. The protocol was approved by the local Ethics Committee of Animal experiments and the Landesamt für Natur, Umwelt und Verbraucherschutz of North Rhine-Westphalia in Germany (reference number: 84-02.04.2015.A199). Six to eight weeks old, genetically engineered, male and female mice with C57BL/6J lineage background were anesthetized with Ketamin/Xylazin (100 mg/kg/(body weight) BW i.p./0.5 mg/kg/BW i.p.) and 1 × 10^7^ pfu Adeno-Cre was applied intratracheally [14]. Viral vectors were provided by the University of Iowa Viral Vector Core (http://www.medicine.uiowa.edu/vectorcore). Prior to tumor induction, the mice were genotyped. We crossed *Kras* ^LSL-G12V^ mice, commonly called RASLO, which express the *Kras* G12V mutant after Cre-recombinase exposure [13] (Supplementary Figure S1a) with the conditional Cre-inducible *Notch1* knock-out model (*Notch1* ^tm1Agt^, commonly called *Notch1* ^fl/fl^) [15] (Supplementary Figure S1b), kindly provided by Freddy Radtke and colleagues. Eight weeks after Cre-application, mice were sacrificed by cervical dislocation and lung tissue was harvested and formalin fixed and paraffin embedded (FFPE). In total, seven mice per group have been analyzed and are indicated as follows: R+ N1w/w (*n* = 7) harboring mutated *Kras* driven tumors with wild type *Notch1* alleles, R+ N1f/w (*n* = 7) harboring mutated *Kras* driven tumors with heterozygous *Notch1* knock-out and R+ N1f/f (*n* = 7) harboring mutated *Kras* driven tumors with homozygous *Notch1* knock-out.

### Genotyping

Genotyping of mice was performed using PCR (Supplementary Figure S1c) in 25 µl total volume according to the manufacturer’s protocol (New England Biolabs, Frankfurt, Germany) using 2 µl of DNA, 0.2 µl Hot Start Taq polymerase (5000 U/ml) 2.5 µl Standard Taq Reaction buffer (10x), 0.5 µl dNTPs (10 mM Mix), 0.5 µl Primer fwd (10 µM), 0.5 µl Primer rev (10 µM), and 18.8 µl DNase/RNase free water. The following primers were used: Ras-fwd 5′CAG​TGC​AAT​GAG​GGA​CCA​GT3′, Ras-rev 5′CAC​CCT​GTC​TTG​TCT​TTG​CTG​ATG3′, Notch1-fwd 5′ CTG​AGG​CCT​AGA​GCC​TTG​AA3′ and Notch1-rev 5′TGT​GGG​ACC​CAG​AAG​TTA​GG3′. Eartag material was lyzed using 100 µl lysis buffer (25 mM NaOH, 0.5 mM EDTA) at 95°C for 1 h and neutralized using 100 µl 40 mM Tris-HCl. The Notch1 PCR indicates whether both Notch1 alleles were floxed (500 bp product) as prerequisite for conditional Notch1 knock-out. The wild type allele which was not floxed revealed a 445 bp product. Thus, in a Notch1 flox/wild type condition, a double band was produced (see control R+ N1f/w). The Ras PCR (280 bp) detected the *Kras* ^LSL-G12V^ expression construct enabling tumor induction. Excision of the loxP flanked stop cassette was mediated by intratracheal inhalation of adenoviral Cre-recombinase as described above.

### Immunohistochemistry

Three micrometre sections from FFPE lung tissue, were deparaffinized and treated according to standard protocols of the routine diagnostics pipeline (Institute for Pathology, University Hospital Cologne, Germany). Staining was performed using hematoxylin & eosine (H&E) and primary antibodies against KI67 (SP6, 1:100, Cell Marque, Rocklin, United States), NICD1 (ab8925, 1:50, Abcam, Cambridge, United Kingdom), SPC (AB3786, 1:100, Millipore, Burlington, United States), CC10 (07-623, 1:250, Millipore) and TAZ (H-70, 1:100, Santa Cruz Biotechnology, Dallas, United States) and the secondary Histofine Simple Stain antibody detection kit (Medac, Wedel, Germany). The LabVision Autostainer 480S (Thermo Fisher Scientific, Waltham, United States) was used for visualization and slides were scanned by the Pannoramic 250 slide scanner (3D Histech, Budapest, Hungary). Murine tumors were analyzed according to the recommendations of mouse models of the human cancers consortium [16]. Protrusions from bronchiolar lining and clear bronchiolar lumen without protrusions have been determined in R+ N1w/w (*n* = 7), R+ N1f/w (*n* = 7) and R+ N1f/f (*n* = 7) mice by three independent H&E stained transverse sections of the lower lung periphery from each of the FFPE lung biopsies.

TAZ protein expression was scored by IHC in murine tumor samples and in clinical samples combined in tissue micro arrays (TMAs). Thirty-eight squamous cell carcinomas (SCC), 37 adenocarcinomas (ADC) and 32 small cell carcinomas (SCLC) of patients have been investigated. TAZ expression in the nucleus or the cytoplasm was rated as positive if more than 10% of tumor cells exhibited expression. The TAZ score was determined by two independent investigators and graded on an integer number four-grade scale (0: no expression, 1: weak expression, 2: moderate expression, 3: strong expression). The TAZ score was obtained by adding the nuclear and the cytoplasmic staining intensity, the nuclear multiplied with 0.75, the cytoplasmic multiplied with 0.25, in order to have a measure of the TAZ activation status [17].

### Cells and Reagents

Lung cancer cell lines were kindly provided by Roman K. Thomas (Department of Translational Genomics, University of Cologne, Germany) and Reinhard Büttner (Institute for Pathology, University Hospital Cologne, Germany). Cells were cultivated in RPMI medium (Life Technologies, Carlsbad, United States) supplemented with 10% FCS (PAA laboratories, Cölbe, Germany). WWTR1/TAZ and NOTCH1 siRNAs (100 nM, Santa Cruz Biotechnology) were transfected using Lipofectamine 3000 (Life Technologies). Plasmid transfection and vector control were performed as previously described [18]. pTight-hASCL1-N174 [19] was a gift from Jerry Crabtree (Addgene plasmid #31876; http://n2t.net/addgene:31876; RRID:Addgene_31876). Cell proliferation was determined by Cell Proliferation kit I [(MTT) assay (Hoffmann-La Roche, Basel, Switzerland)]. MTT assays were performed in 96-well plates with 3,000 cells per well. Cells have been seeded after validated TAZ knock-down in medium containing tetrazolium salt, the reagent which enables the colorimetric detection of proliferating cells. Tetrazolium salt is cleaved to the water insoluble dye formazan by succinate-tetrazolium reductase system. This enzyme is only active in viable cells. The cell proliferation was determined after 24, 48 and 72 h, by measuring the absorbances at A550/690 nm using a plate reader. The protocol was performed according to the manufacturer’s instructions.

For the generation of cell pellet blocks, 1 × 10^7^ cells were centrifuged and fixed in 4% paraformaldehyde (PFA) over night. PFA was removed by centrifugation for 15 min at 2400 rpm and cells were washed with 2 ml 96% ethanol with three drops of glycerin. Cells were again centrifuged for 15 min at 2400 rpm and transferred to tissue capsules and were further processed for paraffin embedding by standard FFPE biopsy protocol.

### qRT-PCR

One microgram of total RNA were transcribed into cDNA using the Super Script™ III, H Reverse transcriptase and Oligo(dt)12-18 Primer (Life Technologies). SYBR Green (Qiagen, Hilden, Germany) was used for qRT-PCR. Used primers were directed against *NOTCH1* (forward 5′-GTC​AAC​GCC​GTA​GAT​GAC​C-3′ and reverse 5′- TTG​TTA​GCC​CCG​TTC​TTC​AG-3′), *ASCL1* (forward 5′-GCA​TGG​AAA​GCT​CTG​CCA​AGA-3′ and reverse 5′- TGG​CAA​AGA​AAC​AGG​CTG​CG-3′), *WWTR1*/TAZ (forward 5′-GAA​GTC​CAT​CCC​CTT​CTG​GT-3′ and reverse 5′-CAA​GCA​GAG​AAT​GAG​GGG​AA-3′) and *GAPDH* (forward 5′-ACT​GCC​AAC​GTG​TCA​GTG​GT-3′ and reverse 5′-GTC​AAA​GGT​GGA​GGA​GTG​G-3′). Primers were derived from the qPrimerDepot of Wenwu Cui and synthetized by Sigma-Aldrich. The 7900HT Fast Real-Time PCR System (Applied Biosystems, Foster City, United States) and the SDS2.2 software (Applied Biosystems) were used. Expression levels were calculated using ∆∆CT method.

### Ethics

Clinical samples were acquired and analyzed as part of routine diagnostic procedures (Institute of Pathology, University Hospital Cologne, Germany) according to the guidelines and with approval of the local ethics committee (reference number: 13-091).

In addition, the datasets analyzed during the study are available in the TCGA repository, accessed by cBioportal [20, 21] webpage https://www.cbioportal.org/. Particularly, a data set of 230 lung ADC samples [22] was accessible under: https://www.cbioportal.org/results/oncoprint?Action=Submit&RPPA_SCORE_THRESHOLD=2.0&Z_SCORE_THRESHOLD=2.0&cancer_study_list=luad_tcga_pub&case_set_id=luad_tcga_pub_3way_complete&data_priority=0&gene_list=NOTCH1%2520KRAS&geneset_list=%20&genetic_profile_ids_PROFILE_COPY_NUMBER_ALTERATION=luad_tcga_pub_gistic&genetic_profile_ids_PROFILE_MUTATION_EXTENDED=luad_tcga_pub_mutations&profileFilter=0&tab_index=tab_visualize.

Moreover, a dataset of 178 lung SCC samples [23] was used, accessible under: https://www.cbioportal.org/results/oncoprint?Action=Submit&RPPA_SCORE_THRESHOLD=2.0&Z_SCORE_THRESHOLD=2.0&cancer_study_list=lusc_tcga_pub&case_set_id=lusc_tcga_pub_cnaseq&data_priority=0&gene_list=NOTCH1%2520KRAS&geneset_list=%20&genetic_profile_ids_PROFILE_COPY_NUMBER_ALTERATION=lusc_tcga_pub_gistic&genetic_profile_ids_PROFILE_MUTATION_EXTENDED=lusc_tcga_pub_mutations&profileFilter=0&tab_index=tab_visualize.

The datasets are available in the TCGA repository, accessed by cBioportal [20, 21] webpage https://www.cbioportal.org/.

### Statistics

Statistical analysis was performed with Graph Pad Prism (STATCON, Witzenhausen, Germany) and SPSS (IBM Corp., Armonk, United States). The two-sided Student’s t-test and the Pearson Correlation Coefficient were used to analyze data for significant differences and correlations. Error bars indicate standard error of the mean (SEM). *p*-values <0.05 were regarded as significant and indicated in the figures as follows: *p ≤ 0.05, **p ≤ 0.01, ***p ≤ 0.001.

## Results

### Conditional Notch1 Deficiency Significantly Increases Tumor Cell Accumulation Inside the Bronchiolar Lumen in a Mutated *Kras* Driven Lung Cancer Mouse Model

**FIGURE 1:**
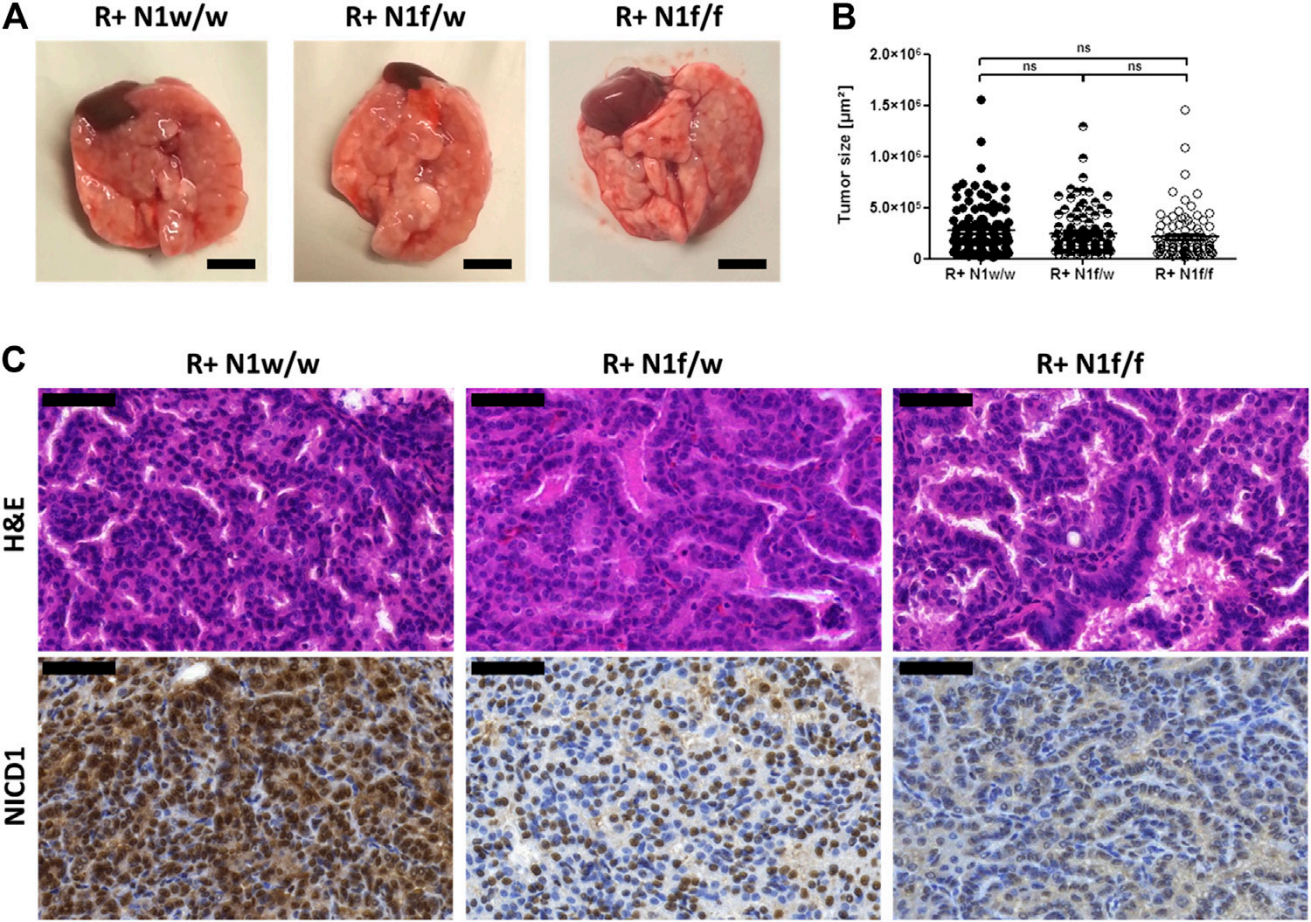
Conditional *Notch1* knock-out in mutated *Kras* driven ADCs induces a columnar papillary morphology. **(A)** Macroscopic view of primary lung tumor tissue harvested eight weeks post adenoviral Cre-recombinase application. Bars indicate 500 µm. **(B)** Three representative H&E stained sections of FFPE lung tissue were scanned and tumor lesions are measured using the Pannoramic Viewer. R+ N1w/w (*n* = 118), R+ N1f/w (*n* = 97), R+ N1f/f (*n* = 91). Statistical analysis was performed using Student’s t-test (ns—not significant; *p < 0.05; **p < 0.01, ***p < 0.001, error bars indicate SEM). **(C)** Microscopic view at ×40 magnification of H&E and NICD1 stain. Bars indicate 50 µm.

Mice, carrying the conditional *Kras* ^LSL-G12V^ expression construct [13] were crossed with conditional *Notch1* knock-out mice [15]. Hence, both, the expression of the *Kras* mutant and the *Notch1* knock-out depend on adenoviral Cre-recombinase application. Eight weeks after intratracheal inhalation of adenoviral Cre-recombinase, lung tissues were harvested (Figure 1A). The size of single tumor lesions was determined and tumors were analyzed histologically (Figure 1B). Immunohistochemical analysis of the intracellular domain of Notch1 (NICD1), shown to be the active Notch1 signaling component, confirmed that *Notch1* was efficiently knocked out in the *Kras* mutant derived tumors (Figure 1C). There was no significant difference in tumor size observed between the mutated *Kras* driven tumors with wild type *Notch1* alleles (R+ N1w/w), the mutated *Kras* driven tumors with heterozygous *Notch1* knock-out (R+ N1f/w) and the mutated *Kras* driven tumors with homozygous *Notch1* knock-out (R+ N1f/f).

Morphologically, the tumors presented as papillary ADCs and showed cuboidal cell shape in R+ N1w/w mice harboring active Notch1 signaling. Notch1 deficiency resulted in a mosaic-like pattern and complete loss of NICD1 in R+ N1f/w and R+ N1f/f mice, resulted in papillary ADCs comprised of cuboidal and columnar tumor cells, respectively (Figure 1C).

**FIGURE 2:**
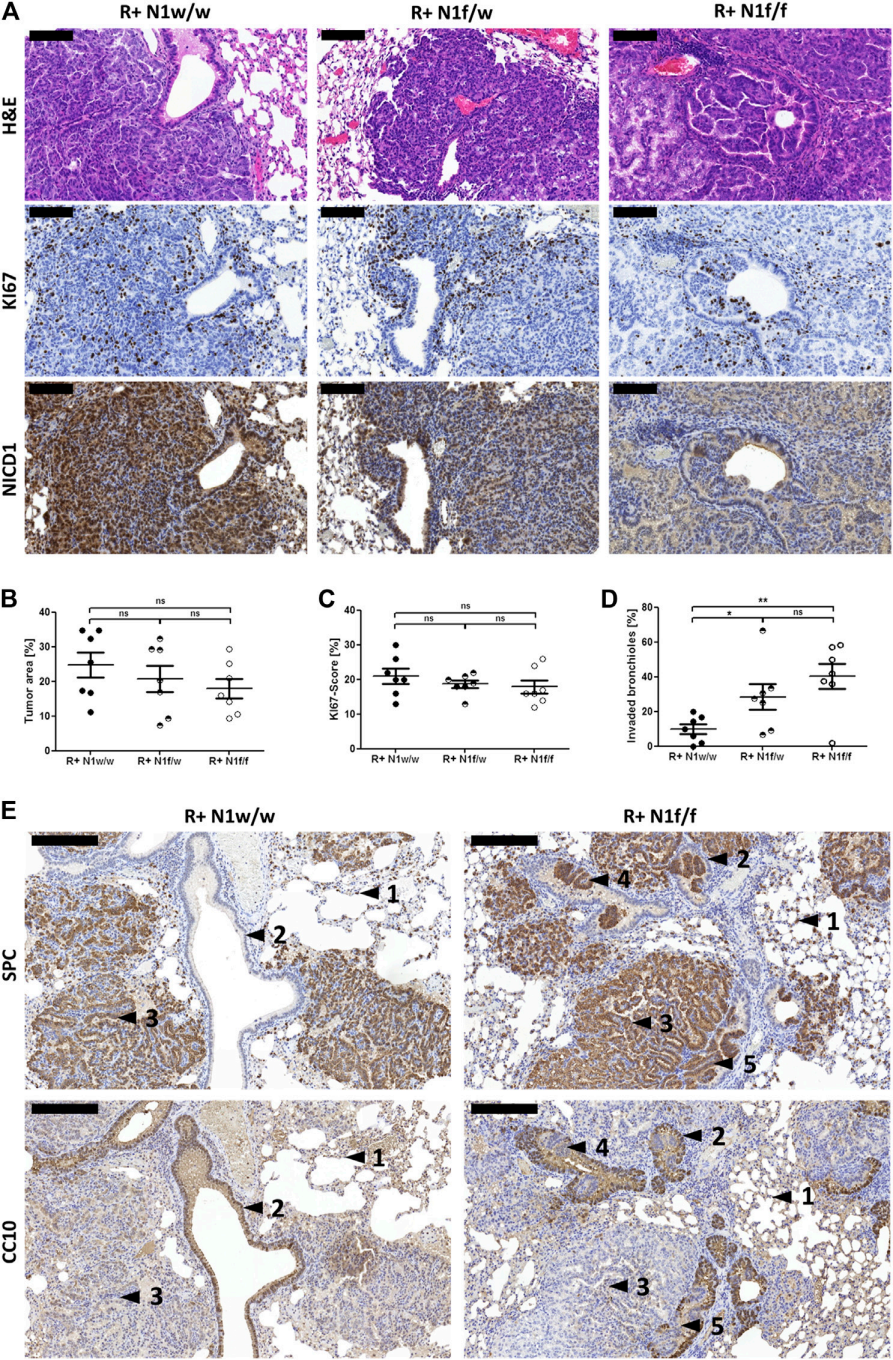
Conditional *Notch1* knock-out in mutated *Kras* driven ADCs induces protrusion from the bronchiolar wall. **(A)** Microscopic view at ×20 magnification of H&E and stains of KI67 and NICD1. Bars indicate 100 µm. **(B)** Three different H&E stained sections from each of the FFPE lung biopsies were scanned and tumor areas were analyzed using the Pannoramic Viewer. **(C)** KI67 stained sections of FFPE lung tissue were scanned and three squares of 100 cells were counted using the Pannoramic Viewer. **(D)** All sections were analyzed to determine tumor buds in the bronchiolar lumen. Protrusions from bronchiolar lining and clear bronchiolar lumen without protrusions of R+ N1w/w (*n* = 7), R+ N1f/w (*n* = 7) and R+ N1f/f (*n* = 7) mice were counted. Statistical analysis was performed using Student’s t-test (ns—not significant; *p < 0.05; **p < 0.01, ***p < 0.001, error bars indicate SEM). **(E)** Microscopic view at ×10 magnification of SPC and CC10 stain. Bars indicate 200 µm. Findings are marked by black arrows: 1) normal SPC+ cells, 2) normal CC10+ cells, 3) ADCs composed of SPC+ tumor cells, 4) SPC+ cells protruding from the bronchiolar lining, 5) SPC+ cells forming tumor buds.

The determined tumor area upon *Notch1* knock-out was slightly decreased from 24.68 to 17.90% (*p* = 0.1672) as well as tumor cell proliferation, indicated by the KI67 index, from 21.00 to 17.86% (*p* = 0.3034) in a dose-dependent manner, though not significantly, comparing R+ N1w/w and R+ N1f/f mice (Figures 2A–C). Intriguingly, tumor cell accumulation inside the bronchiolar lumen with proliferating cells which protrude from the bronchiolar lining, was significantly increased both upon the loss of one *Notch1* allele [from 9.82 to 28.40% (*p* = 0.0390)] and the loss of two *Notch1* alleles [from 9.82 to 40.34% (*p* = 0.0020)] (Figure 2D). The cell lineage markers, Surfactant Protein C (SPC) and Clara Cell 10 KDa Secretory Protein (CC10) were used to identify the differentiation of dysplastic cells accumulations inside the bronchiolar space. SPC+ cells representing alveolar type II cells, were located in the alveolar periphery (arrow 1) and CC10 + Clara cells lined the bronchiolar walls (arrow 2). ADCs which grew predominantly peri-bronchiolarly were composed of SPC+ tumor cells (arrow 3). In R+ N1w/w mice, bronchiolar airways were rarely invaded by SPC+ tumor cells whereas in R+ N1f/f mice, SPC+ cells which were located within the bronchiolar lining formed epithelial carcinoma *in situ* lesions protruding from the bronchiolar wall (arrow 4) and budding into the bronchiolar lumen (arrow 5) (Figure 2E).

Taken together, these data suggest that conditional Notch1 deficiency in this autochthonous *Kras* ^LSL-G12V^ driven lung cancer mouse model induces papillary ADCs accompanied by SPC+ tumor cell accumulation inside the bronchiolar lumen, which is not found under Notch1 wild-type conditions.

### Co-Occurrence of Putative Loss-of-Function Mutations in *NOTCH1* with Genomic *KRAS* Aberrations in Lung ADCs

To determine whether Notch1 deficiency due to genomic aberrations can also be observed in human mutated *KRAS* driven ADCs, we used TCGA database and investigated the publicly available lung ADC dataset containing 230 cases [22] for genomic aberrations in *KRAS* and *NOTCH1*. We found ten genomic alterations in *NOTCH1* (4.3%) (Table 1), of which five were putative loss-of-function aberrations (2.15%). One of the *NOTCH1* alteration was annotated as an oncogenic likely loss-of-function frame shift insertion. The other four of the *NOTCH1* alterations were missense mutations in domains coding for the extracellular EGF-like repeats, which may result in interference with Notch ligand binding and reduce NOTCH1 signaling [18]. In four out of five cases harboring putative *NOTCH1* loss-of-function alteration, *KRAS* was also altered by genomic amplification and mutation (Table 1). In 178 cases of SCCs obtained from TCGA database [23], genomic aberrations in NOTCH1 and KRAS are mutually exclusive and do not co-occur (Supplementary Table S1).

**TABLE 1:** Putative loss-of-function *NOTCH1* aberrations co-occur with *KRAS* mutations and genomic amplifications in human lung ADCs. A publicly available TCGA dataset provided 230 cases of lung ADCs [22] including the five listed cases which harbor putative loss-of-function genomic alterations in *NOTCH1*. *KRAS* alterations are listed accordingly.

Case	Putative loss-of-function *NOTCH1* alteration	Genomic *KRAS* alteration
TCGA-44-2657	E256Q	Amplification
TCGA-44-7672	D259N	Amplification + G12A
TCGA-50-5931	D297G	Not altered
TCGA-55-1592	D1815Gfs*19	Amplification
TCGA-67-3774	P820L	Amplification + G12F

Taken together, this data suggests that Notch1 deficiency by putative inactivating *NOTCH1* alterations occurs together with *KRAS* aberrations particularly in human ADCs of the lung, thus giving clinical importance to our findings.

### Deletion of *Notch1* Triggered TAZ Expression in Mutated *Kras* ^LSL-G12V^ Driven Lung Cancer

Since TAZ and Notch signaling cooperate in epidermal cell fate decisions [10], we aimed to decipher whether upon *Notch1* deletion TAZ expression is altered in mutated *Kras* driven ADCs. ASCL1 is a master regulator in Notch signaling [18] and is used as a positive control for Notch1 deficiency in the following analyses.

**FIGURE 3:**
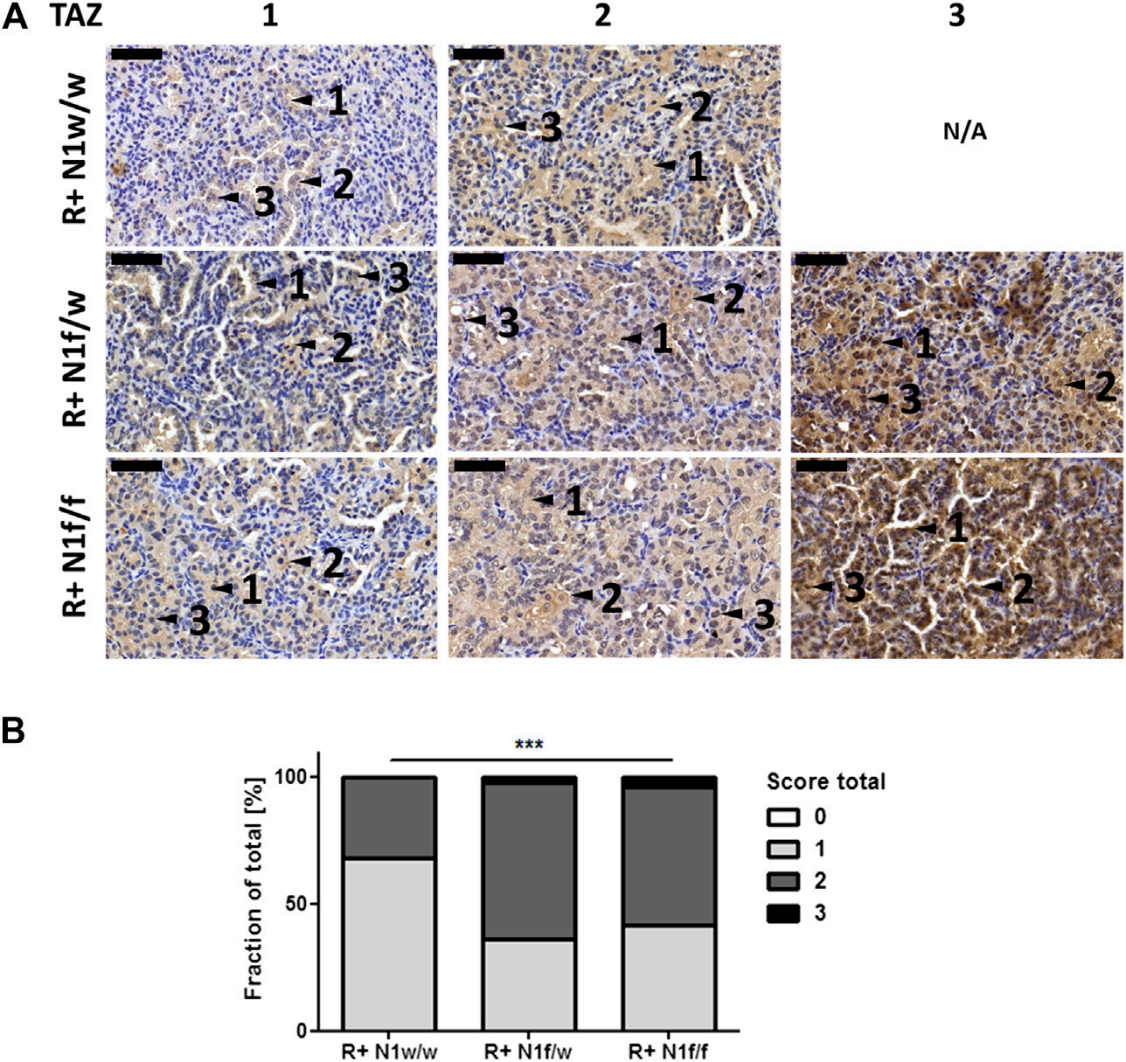
Increased TAZ expression is associated to *Notch1* deletion in mutated *Kras* driven ADCs. **(A)** Microscopic view at ×40 magnification of TAZ stains of FFPE lung tissue. Bars indicate 50 µm. Findings are marked by black arrows: 1) cytoplasmic expression, 2) membranous expression, 3) nuclear expression. na—not available. **(B)** TAZ stained sections were scanned and analyzed using the Pannoramic Viewer. The total score of TAZ protein expression was determined from three representative mice per group per single lung lesion with 0 (no expression), 1 (weak expression), 2 (moderate expression), 3 (strong expression). R+ N1w/w (*n* = 49), R+ N1f/w (*n* = 47), R+ N1f/f (*n* = 47). Statistical analysis was performed using Student’s *t*-test (ns—not significant; *p < 0.05; **p < 0.01, ***p < 0.001, error bars indicate SEM).

We investigated TAZ expression in R+ N1w/w, R+ N1f/w and R+ N1f/f mice by IHC and determined a total protein expression score per single lesion. We found that partial and full *Notch1* knock-out in mutated *Kras* driven ADCs increased TAZ expression significantly (*t*-test, two-sided, *p* = 0.0012). In addition, Pearson Correlation analysis revealed a weak correlation of moderate and high TAZ expression to partial and full *Notch1* knock-out R = 0.27231; *p* = 0.00029) (Figure 3). Similar results were obtained *in vitro* using the ADC cell line PC9 which has *NOTCH1* expression and low levels of *ASCL1* (Supplementary Figure S2a). ASCL1 is negatively regulated by NOTCH1 [18]. Thus, PC9 cells showed increased *ASCL1* and *WWTR1*/TAZ expression upon *NOTCH1* knock-down (Supplementary Figure S2b). Upon plasmid derived ASCL1 expression in PC9 cell clones we detected *WWTR1*/TAZ up-regulation, as well (Supplementary Figure S2c). These data show that TAZ expression is regulated by Notch1.

**FIGURE 4:**
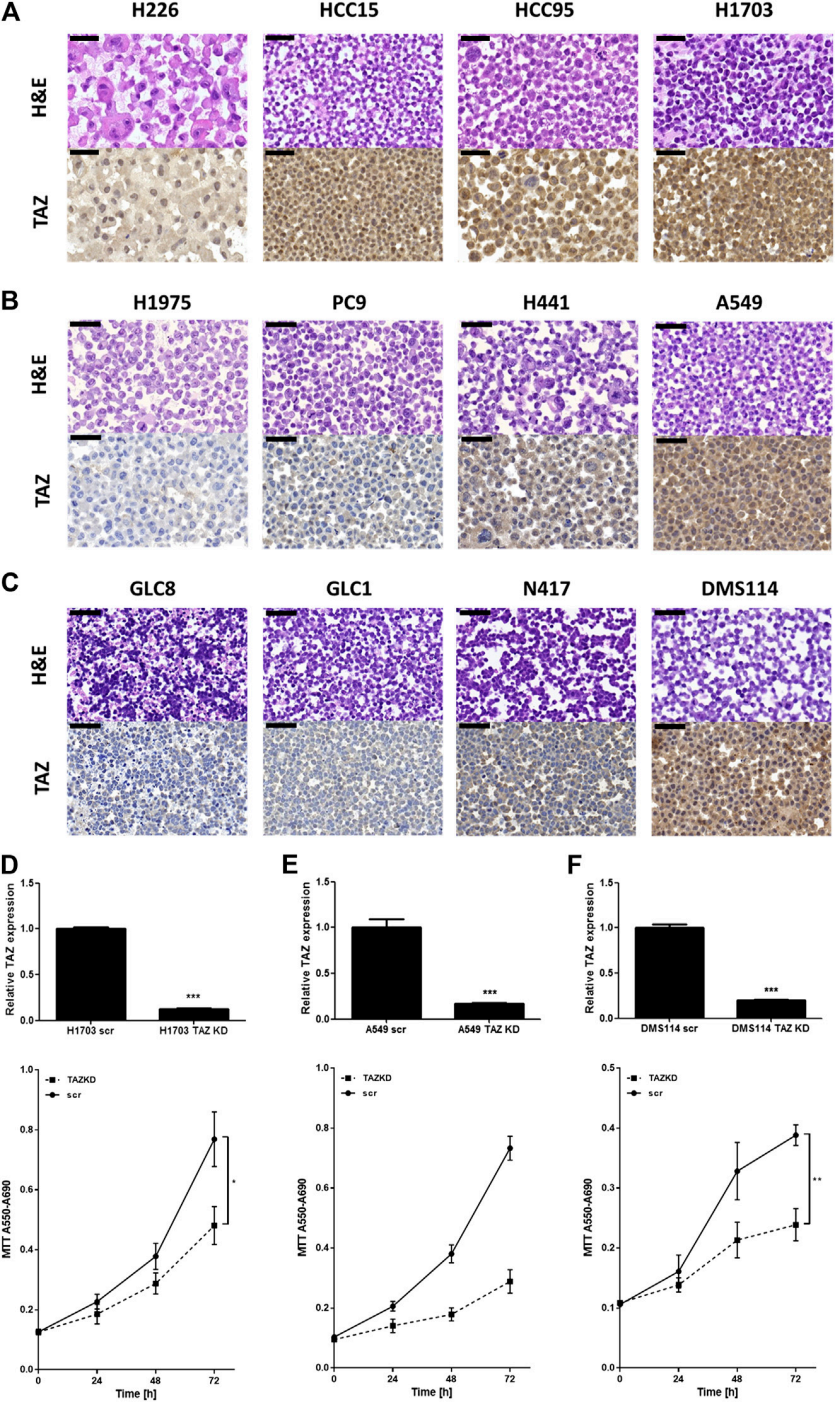
*WWTR1*/TAZ knock-down reduces cell proliferation in SCC, ADC and SCLC cells. **(A–C)** Cell lines have been prepared in FFPE cell blocks for microscopic analysis. The microscopic view at ×40 magnification of H&E and TAZ IHC stain of four SCC cell lines (H226, HCC15, HCC95, H1703), ADC cell lines (H1975, PC9, H441, A549) and SCLC cell lines (GLC8, GLC1, N417, DMS114), respectively. Bars indicate 50 µm. **(D–F)** Upper: Relative TAZ mRNA expression was determined by qRT-PCR 24 h after transfection (*n* = 4) normalized to GAPDH housekeeper using ΔΔCT method. Lower: MTT assay for measuring cell proliferation by absorbance at A550–A690 nm wavelength 0, 24, 48 and 72 h after WWTR1/TAZ knock-down (*n* = 4). Statistical analysis was performed using Student’s *t*-test (ns—not significant; *p < 0.05; **p < 0.01, ***p < 0.001, error bars indicate SEM). **(D)** SCC cell line H1703; **(E)** ADC cell line A549; **(F)** SCLC cell line DMS114.

Furthermore, we investigated TAZ expression in cell blocks of human lung carcinoma cell lines, representing the most frequent lung carcinoma entities, including four SCC cell lines (H226, HCC15, HCC95, H1703), four ADC cell lines (H1975, PC9, H441, A549) and four SCLC cell lines (GLC8, GLC1, N417, DMS114). All SCC cell lines exhibited high TAZ expression, among the ADC cell lines, A549 and H441 cells, both harboring *KRAS* mutations, showed the highest TAZ expression. Among the SCLC cell lines only DMS114 cells showed TAZ expression (Figures 4A–C). Knock-down of TAZ in H1703, A549 and DMS114 cells using three pooled target specific siRNAs significantly reduced tumor cell proliferation after 72 h determined by MTT assay (Figures 4D–F). In the cell lines GLC8 and GLC1, that showed only marginal TAZ expression, TAZ knock-down did not affect tumor cell proliferation (Supplementary Figure S3). Thus, we hypothesize that TAZ protein expression correlates to the inhibitory effect of TAZ knock-down on cell proliferation and that TAZ protein expression might serve as a predictive marker for treatment with TAZ signaling inhibitors.

**FIGURE 5:**
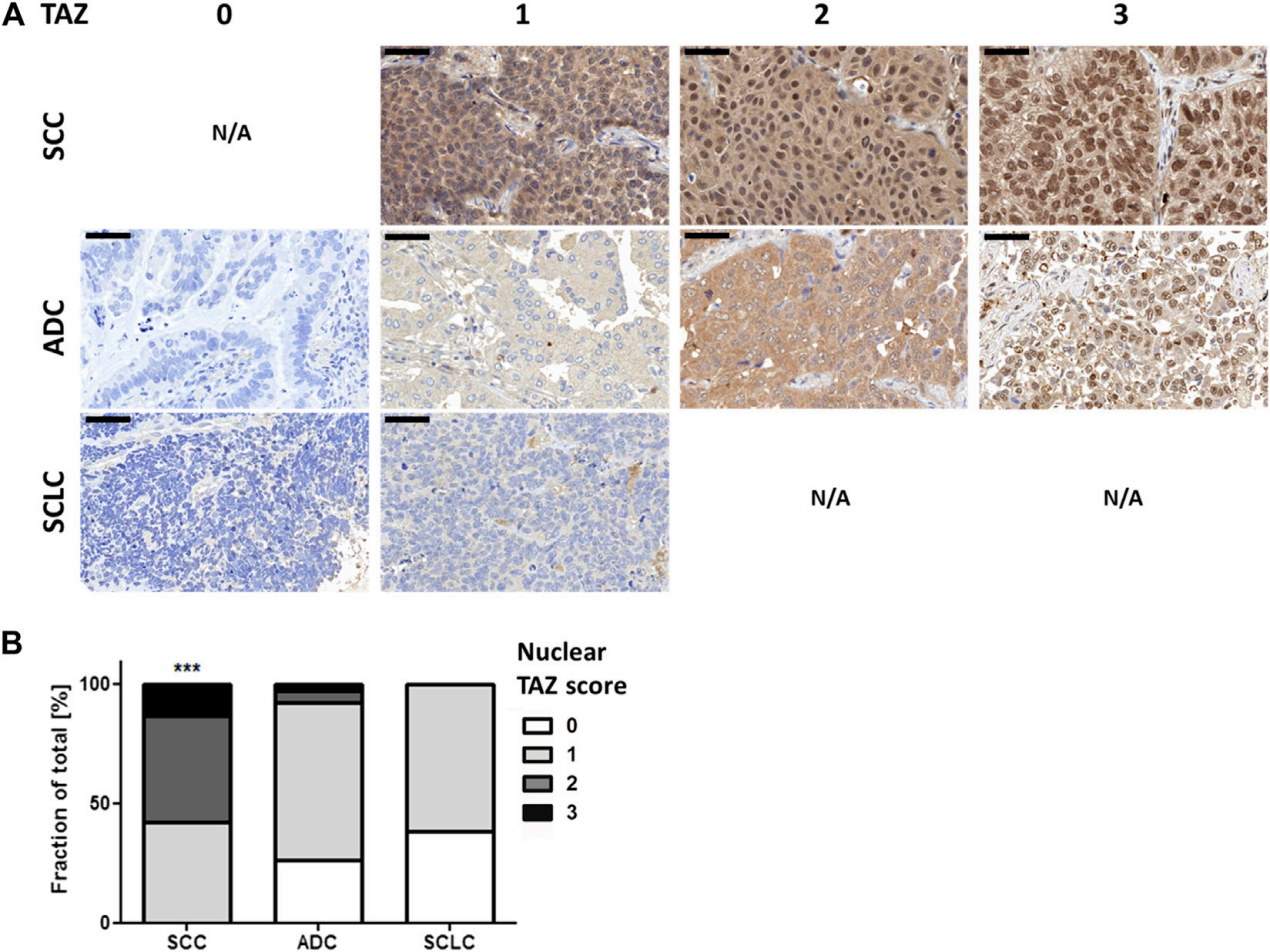
Nuclear TAZ protein expression is associated to SCC. Tissue micro arrays comprising SCC (*n* = 38), ADC (*n* = 37) and SCLC (*n* = 32) were stained for TAZ expression by IHC. **(A)** Microscopic view at ×40 magnification. Bars indicate 50 µm. N/A—not available. **(B)** TAZ stained sections were scanned, analyzed using the Pannoramic Viewer and scored in terms of TAZ nuclear localization with 0 (no expression), 1 (weak expression), 2 (moderate expression), 3 (strong expression). Statistical analysis was performed using Pearson Correlation (ns—not significant; *p < 0.05; **p < 0.01, ***p < 0.001).

To show whether this TAZ distribution also applies to clinical patient samples, we investigated TAZ expression in lung carcinoma samples acquired during routine diagnostics. Patients’ lung SCCs, ADCs and SCLCs were analyzed by IHC using TMAs with regard to the increased TAZ expression and nuclear TAZ localization (Figure 5; Table 2; Supplementary Figure S4) because the nuclear translocation of TAZ indicates activation of the Hippo signaling pathway [24]. Within the clinicopathologic parameters we found no association of TAZ expression to age and gender (Table 2).

**TABLE 2:** Clinicopathological characteristics. Histology, age at diagnosis and gender are correlated to the TAZ expression in the nucleus. Biopsy samples have been combined on tissue micro arrays comprising SCC (*n* = 38), ADC (*n* = 37) and SCLC (*n* = 32). Statistical analysis was performed using Pearson Correlation. R-values and *p*-values are indicated.

TAZ expression in the nucleus							
		0	1	2	3	R (Pearson)	*p*-value
Histology							
	Total (%)	22 (20.6)	60 (56.1)	19 (17.7)	6 (5.6)	0.5999	<0.0001
	SCC	0	16	17	5		
	ADC	10	24	2	1		
	SCLC	12	20	0	0		
Age at diagnosis (years)							
< 65	Total	13 (12.1)	26 (24.3)	5 (4.7)	3 (2.8)	−0.0718	0.467397
	SCC	0	8	5	3		
	ADC	5	9	0	0		
	SCLC	8	9	0	0		
≥65	Total	9 (8.4)	34 (31.8)	14 (13.1)	3 (2.8)		
	SCC	0	8	12	2		
	ADC	5	15	2	1		
	SCLC	4	11	0	0		
Gender							
Male	Total	13 (12.1)	40 (37.4)	16 (15.0)	4 (3.7)	0.1075	0.270407
	SCC	0	11	14	4		
	ADC	7	18	2	0		
	SCLC	6	11	0	0		
Female	Total	9 (8.4)	20 (18.7)	3 (2.8)	2 (1.9)		
	SCC	0	6	2	1		
	ADC	3	5	1	1		
	SCLC	6	9	0	0		

Importantly, high total and nuclear TAZ protein expression was detected in ADCs, associated to the SCC entity using Pearson Correlation (R_total_ = 0.66464; R_nucleus_ = 0.5999; *p* < 0.0001), but less present in the SCLC cohort (Figures 5C, D; Table 2). Addressing TAZ expression and survival in publicly available TCGA data [22, 23], WWTR1/TAZ amplification served as a negative prognostic factor in ADCs (Logrank test; *p* = 0.0533), but had no impact on survival in SCCs (Logrank test; *p* = 0.988) (Supplementary Figure S5).

This data demonstrates that TAZ expression is found in SCCs, ADCs and SCLCs of the lung, serves as a negative prognostic factor in ADCs and is mainly associated to the SCC entity. Interestingly, TAZ expression which is related to a poor prognosis in ADCs, might be regulated by NOTCH1 deficiency.

## Discussion

In this study, we investigated the effects of functional Notch1 deficiency in an autochthonous *Kras* ^LSL-G12V^ driven lung adenocarcinoma mouse model.

Mutated *Kras* driven murine lung tumors are considered to be weakly, if at all, invasive and it has been discussed that additional deletion of *Tp53* is required for a full stromal invasive growth [14, 25]. An alternative highly aggressive pathway for invasion in humans involves the spread of tumor cells via air spaces (STAS), for example presenting with micropapillary growth pattern associated with poor survival probability [26, 27].

Spread through air spaces is thought to involve tumor budding at the invasive front of tumors [28] and we also observed tumor cells accumulating inside the bronchiolar lumen. Mutated *Kras* driven tumors generally arise in the periphery of the lung and are less frequently observed in the central major airways which is thought to be due to the cellular origin of lung tumors [27].

SPC+ alveolar type II cells and CC10+ SPC+ bronchioalveolar stem cells are suggested to be the major cell type of lung cancer origin in humans and mice [6, 7, 27]. In line with this notion, the tumors observed in the *Kras* driven lung cancer mouse model used here, positively stain for SPC, indicating canonical pathophysiological tumorigenesis with respect to the cell of origin.

Carcinogenesis in lung cancer, like in many other solid tumors, has been shown to occur through a multistep process [29]. In the used lung cancer mouse model, the activating *Kras* G12V mutation induce an adenomatous change in the lung parenchyma because the proliferating cells are not eliminated. Interestingly, a similar mouse model with *Kras* activation in the gut does not lead to tumor formation since the proliferating cells are just shed, unless an inactivating genomic aberration in *adenomatous polyposis coli* (*Apc*) is present in addition [30, 31].

Similarly, in the central airways of the lung, proliferative cells can be easily shed into the lumen and therefore no dysplastic cell mass is able to accumulate, which is the required condition for further mutation hits resulting in a multistep carcinogenesis. Our findings on lung cancer in mice upon Kras activation combined with Notch1 inactivation clearly show an accumulation of tumor cells in the lumen of bronchioles protruding from the bronchiolar epithelium. The enrichment of tumor cells in the bronchiolar lumen is reasoned by Notch1 deficiency on lateral inhibition in the tumor cells [32]. Moreover, the reduced cell contact inhibition, caused by the induced TAZ expression upon Notch1 deficiency followed by the Hippo pathway alteration, facilitates protruding of proliferating tumor cells from the bronchiolar lining [33, 34].

Interestingly, TAZ regulates lineage decisions in mammary epithelium, being active in basal cells, the likely cell of origin of SCCs, and mediating luminal differentiation upon down-regulation [24]. Recently, Totaro and colleagues linked YAP/TAZ and Notch signaling to epidermal cell fate decisions showing that active YAP/TAZ induced Notch inhibition [10], which is in line with our findings.

In addition, Xu and colleagues used a *Kras* ^LSL-G12D^ driven mouse model and crossed this with a dominant negative *Maml1* mutant mouse model. Under healthy conditions Maml1 forms a signaling complex together with Notch, but linked to the dominant negative mutant of *Maml1*, Notch signaling was abrogated completely in the mutated *Kras* driven lung tumors. They identified a squamous cell differentiation in 5–17% of tumor cells accompanied by reduced tumor burden upon deletion of Notch signaling [35].

Moreover, the inactivating *NOTCH* mutations described in human lung tumors have been found mainly in centrally located tumor represented by neuroendocrine differentiated or squamous cell carcinomas [8, 18], which is consistent with the findings in the mouse model described here.

Therefore, we show here for the first time in an *in vivo* model that Notch1 deficiency is able to induce tumor cell accumulation even in central airways in a mutated *Kras* driven lung cancer mouse model, which may represent the early steps of a typical multistep carcinogenesis. Since putative loss-of-function mutations co-occur in a small fraction of lung ADCs with *KRAS* mutations and genomic amplifications, there is a rationale to use this model to test therapy regiments targeting TAZ. Since therapeutic interventions directly blocking TAZ signaling are missing [36], treatments acting upstream and downstream of TAZ signaling might be investigated in this model.

## Conclusion

Our study provides a genetically engineered mouse model to investigate tumorigenesis of autochthonous *Kras* ^LSL-G12V^ driven lung cancer upon conditional *Notch1* knock-out. Particularly, we found that Notch1 deficiency triggered tumor cell accumulation inside the bronchiolar lumen and expression of the Hippo pathway transcription factor TAZ, which may represent a target for cancer therapy in this specific subgroup of lung ADCs.

## Data Availability

The datasets presented in this study can be found in online repositories. The names of the repository/repositories and accession number(s) can be found in the article/Supplementary Material.

## Abbreviations:

ADC, adenocarcinoma; CC10, Clara cell 10 KDa secretory protein; f, floxed allele; FFPE, formalin-fixed paraffin-embedded; IHC, immunohistochemistry; KRAS, Kirsten rat sarcoma viral oncogene homolog; LSL, lox-stop-lox; NICD, notch intracellular domain; NSCLC, non-small cell lung cancer; SCC, squamous cell lung carcinoma; SCLC, small cell lung carcinoma; SPC, surfactant protein C; STAS, spread of tumor cells via air spaces; TAZ, transcriptional coactivator with PDZ-binding motif; TCGA, The Cancer Genome Atlas; TMA, tissue micro-array; w, wild type allele; WWTR1, WW domain containing transcription regulator 1; YAP, yes-associated protein.
