# Supplementary material for: Notch1 Deficiency Induces Tumor Cell Accumulation Inside the Bronchiolar Lumen and Increases TAZ Expression in an Autochthonous Kras LSL-G12V Driven Lung Cancer Mouse Model
Source: Pathol Oncol Res. 2021 Apr 16;27:596522. doi: 10.3389/pore.2021.596522 (PMC8262161; doi:10.3389/pore.2021.596522)
Supplement: Supplementary file 1 [file Table1.docx]

| Case | **Putative**  **loss-of-function**  **NOTCH1 alteration** | **Genomic KRAS alteration** |
| --- | --- | --- |
| TCGA-44-2657 | E256Q | amplification |
| TCGA-44-7672 | D259N | amplification + G12A |
| TCGA-50-5931 | D297G | not altered |
| TCGA-55-1592 | D1815Gfs*19 | amplification |
| TCGA-67-3774 | P820L | amplification + G12F |

**Table 2. Putative loss-of-function *NOTCH1* aberrations co-occur with *KRAS* mutations and genomic amplifications in human lung ADCs.** A publicly available TCGA dataset provided 230 cases of lung ADCs [22] including the five listed cases which harbor putative loss-of-function genomic alterations in *NOTCH1*. *KRAS* alterations are listed accordingly.
