# Supplementary material for: Notch1 Deficiency Induces Tumor Cell Accumulation Inside the Bronchiolar Lumen and Increases TAZ Expression in an Autochthonous Kras LSL-G12V Driven Lung Cancer Mouse Model
Source: Pathol Oncol Res. 2021 Apr 16;27:596522. doi: 10.3389/pore.2021.596522 (PMC8262161; doi:10.3389/pore.2021.596522)
Supplement: Supplementary file 2 [file Table2.docx]

|  | | **TAZ expression in the nucleus** | | | |  | |
| --- | --- | --- | --- | --- | --- | --- | --- |
|  |  | **0** | **1** | **2** | **3** | **R (Pearson)** | **p-value** |
| **Histology** | | | | | | | |
|  | ***Total (%)*** | ***22 (20.6)*** | ***60 (56.1)*** | ***19 (17.7)*** | ***6 (5.6)*** | **0.5999 <0.0001** | |
|  | **SCC** | **0** | **16** | **17** | **5** |  |  |
|  | **ADC** | **10** | **24** | **2** | **1** |  |  |
|  | **SCLC** | **12** | **20** | **0** | **0** |  |  |
| **Age at diagnosis (years)** | | | | | | | |
| **< 65** | ***total*** | ***13 (12.1)*** | ***26 (24.3)*** | ***5 (4.7)*** | ***3 (2.8)*** | **-0.0718 0.467397** | |
|  | **SCC** | **0** | **8** | **5** | **3** |  |  |
|  | **ADC** | **5** | **9** | **0** | **0** |  |  |
|  | **SCLC** | **8** | **9** | **0** | **0** |  |  |
| **≥ 65** | ***total*** | ***9 (8.4)*** | ***34 (31.8)*** | ***14 (13.1)*** | ***3 (2.8)*** |  |  |
|  | **SCC** | **0** | **8** | **12** | **2** |  |  |
|  | **ADC** | **5** | **15** | **2** | **1** |  |  |
|  | **SCLC** | **4** | **11** | **0** | **0** |  |  |
| **Gender** | | | | | | | |
| **male** | ***total*** | ***13 (12.1)*** | ***40 (37.4)*** | ***16 (15.0)*** | ***4 (3.7)*** | **0.1075 0.270407** | |
|  | **SCC** | **0** | **11** | **14** | **4** |  |  |
|  | **ADC** | **7** | **18** | **2** | **0** |  |  |
|  | **SCLC** | **6** | **11** | **0** | **0** |  |  |
| **female** | ***total*** | ***9 (8.4)*** | ***20 (18.7)*** | ***3 (2.8)*** | ***2 (1.9)*** |  |  |
|  | **SCC** | **0** | **6** | **2** | **1** |  |  |
|  | **ADC** | **3** | **5** | **1** | **1** |  |  |
|  | **SCLC** | **6** | **9** | **0** | **0** |  |  |

**Table 1. Clinicopathological characteristics.** SCC histological subtype, age at diagnosis < 65 years at diagnosis and male gender are correlated to the TAZ expression in the nucleus. Biopsy material was combined on tissue micro arrays comprising SCC (n=38), ADC (n=37) and SCLC (n=32). Statistical analysis was performed using Pearson Correlation.
