# Supplementary material for: Notch1 Deficiency Induces Tumor Cell Accumulation Inside the Bronchiolar Lumen and Increases TAZ Expression in an Autochthonous Kras LSL-G12V Driven Lung Cancer Mouse Model
Source: Pathol Oncol Res. 2021 Apr 16;27:596522. doi: 10.3389/pore.2021.596522 (PMC8262161; doi:10.3389/pore.2021.596522)
Supplement: Supplementary file 3 [file DataSheet1.PDF]

Pathology & Oncology Research - Electronic Supplementary Material

Title

Notch1 deficiency induces tumor cell accumulation inside the bronchiolar lumen and increases TAZ expression in an autochthonous *Kras*$^{LSL-G12V}$ driven lung cancer mouse model

Author names and affiliations

Lydia Meder[1,2*], Alexandra Florin[3], Luka Ozretić[4], Marieke Nill[1,2], Mirjam Koker[1,2], Sonja Meemboor[3], Freddy Radtke[5], Linda Diehl[6], Roland T. Ullrich[1,2], Margarete Odenthal[2,3], Reinhard Büttner[2,3], Lukas C. Heukamp[7,8]

[1] Department I of Internal Medicine, University Hospital Cologne, Kerpener Straße 62, 50937 Cologne, Germany

[2] Center for Molecular Medicine Cologne, University of Cologne, Robert-Koch-Straße 21, 50931 Cologne, Germany

[3] Institute for Pathology, University Hospital Cologne, Kerpener Straße 62, 50937 Cologne, Germany

[4] Department of Cellular Pathology, Royal Free Hospital, London NW3 2QG, UK

[5] École Polytechnique Fédérale de Lausanne, Swiss Institute for Experimental Cancer Research, 1015 Lausanne, Switzerland

[6] Institute of Experimental Immunology and Hepatology, University Medical Center Hamburg-Eppendorf, Martinistr. 52, 20246 Hamburg, Germany

[7] Institute for Hematopathology Hamburg, Fangdieckstraße 75a, Hamburg, Germany

[8] Lungen Netzwerk NOWEL, Georgstraße 12, 26121 Oldenburg, Germany

*lydia.meder@uk-koeln.de

Case	Genomic NOTCH1 alteration	Genomic KRAS alteration
TCGA-39-5037-01	D2020H	-
TCGA-18-5592-01	G977*	-
TCGA-22-1016-01	K428*	-
TCGA-22-5491-01	S2471*; P2469S	-
TCGA-33-4583-01	X1215_splice	-
TCGA-51-4079-01	Q2361*	-
TCGA-66-2759-01	S2211*	-
TCGA-66-2793-01	G1165Qfs*13	-
TCGA-66-2795-01	W1075*	-
TCGA-22-5480-01	N390S	-
TCGA-66-2789-01	R353C; C429S	-
TCGA-21-5782-01	V2038L	-
TCGA-22-5485-01	Y550N	-
TCGA-22-5492-01	R1784L	-
TCGA-34-5236-01	G957W	-
TCGA-63-6202-01	-	Q61H
TCGA-21-1078-01	-	C118S
TCGA-37-4135-01	-	amplification
TCGA-43-3394-01	-	amplification
TCGA-60-2698-01	-	amplification
TCGA-66-2767-01	-	amplification

Supplementary Table 1. Putative loss-of-function *NOTCH1* aberrations and *KRAS* mutations and genomic amplifications are mutually exclusive in lung SCCs. A publicly available TCGA dataset providing 178 cases of lung SCCs [23] includes 7.8 % (14/178) of cases harboring putative loss of function aberrations in *NOTCH1* and 3.4% (6/178) of cases harboring genomic alterations in *KRAS*.

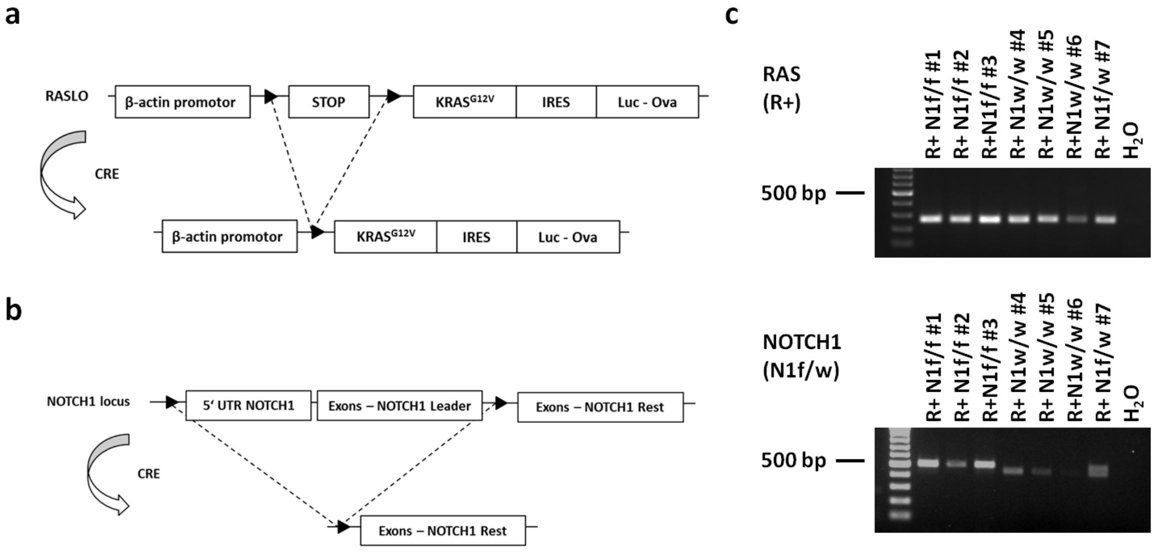

Supplementary Figure 1. Genotyping of genetically engineered mice. (a) In the RASLO construct the *Kras* mutated G12V gene is driven by the ß-actin chicken promoter, but expression is blocked by a stop codon region, which is flanked by loxP sites (black triangles). Upon adenoviral Cre-recombinase application, the stop codon is deleted and the *Kras*$^{LSL\text{-}G12V}$ oncogene is expressed. Due to the fused downstream located internal ribosomal entry site (IRES), the luciferase and ovalalbumin reporters are expressed simultaneously with the *Kras* mutant. (b) The conditional *Notch1* knock-out construct comprises the murine *Notch1* locus encoding the Notch1 receptor. The 5' untranslated region (5'UTR) and the exons encoding the *Notch1* leader peptide responsible for signal induction were flanked by loxP sites (black triangles). Upon adenoviral Cre-recombination, this region is deleted and only truncated non-functional Notch1 rest peptide is encoded. (c) PCR results of the Ras PCR (280 bp) detecting the *Kras*$^{LSL\text{-}G12V}$ expression construct and of the *Notch1* PCR detecting deleted/"floxed" (f) *Notch1* alleles with 500 bp and wild-type (w) *Notch1* alleles with 445 bp.

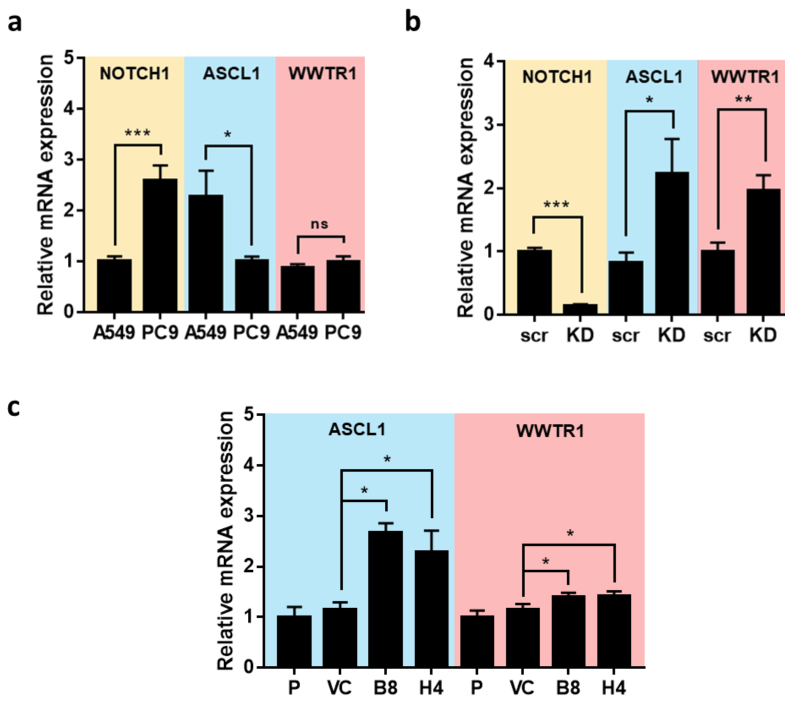

Supplementary Figure 2. NOTCH1 knock-down and ASCL1 expression increase WWTR1/TAZ expression. (a) Relative mRNA expressions of NOTCH1, ASCL1 and WWTR1 of unstimulated A549 and PC9 cells are determined by qRT-PCR. (b) Relative mRNA expressions of NOTCH1, ASCL1 and WWTR1 of PC9 cells, 48 h after transfection with scrambled and NOTCH1 siRNA knock-down (KD) are determined by qRT-PCR. (c) Relative mRNA expressions of ASCL1 and WWTR1 of PC9 cells (P, parental) are determined by qRT-PCR, after transfection with Vector Control (VC) or stably selected clones transfected with an ASCL1 expression plasmid. All expression values are calculated by the ΔΔCT-method. Statistical analysis was performed using Student's t-test (ns – not significant; * p <0.05; ** p <0.01, ** p <0.001, error bars indicate SEM).

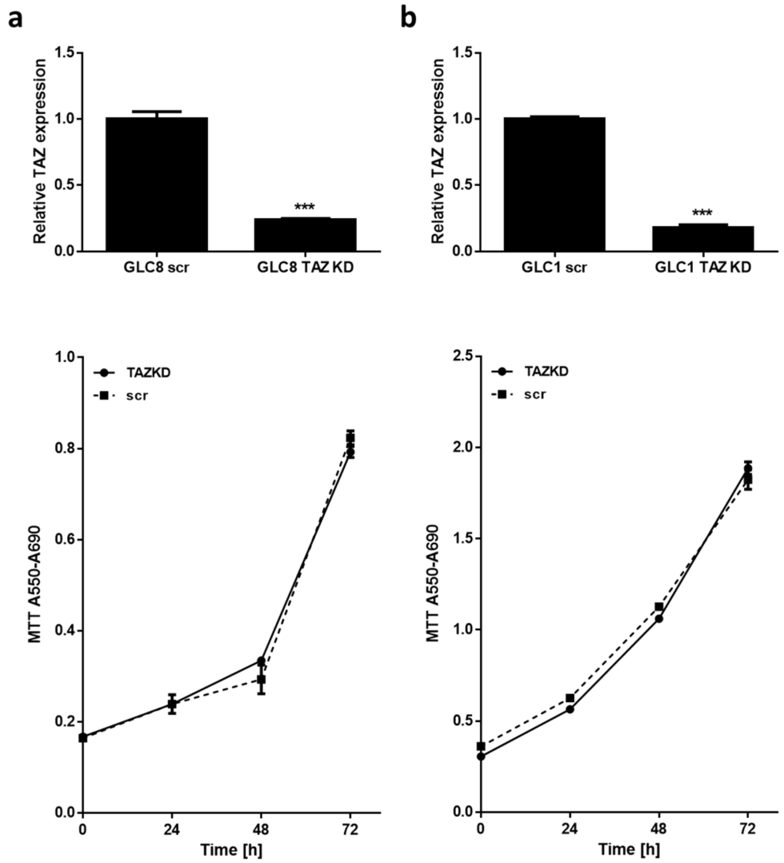

Supplementary Figure 3. The inhibitory proliferative effect of WWTR1/TAZ knock-down is dependent in TAZ protein expression. (a+b) Cell proliferation was analyzed by MTT assay in the SCLC cell lines GLC8 and GLC1 cells, harboring marginal TAZ protein expression. Upper: Relative TAZ mRNA expression was determined by qRT-PCR 24 h after transfection (n=4) normalized to GAPDH housekeeper using ΔΔCT method. Lower: Representative MTT assay for measuring cell proliferation by absorbance at A550-A690 nm wavelength 0 h, 24 h, 48 h and 72 h after WWTR1/TAZ knock-down (n=4). Statistical analysis was performed using Student's t-test (ns – not significant; * $p < 0.05$; ** $p < 0.01$, *** $p < 0.001$, error bars indicate SEM).

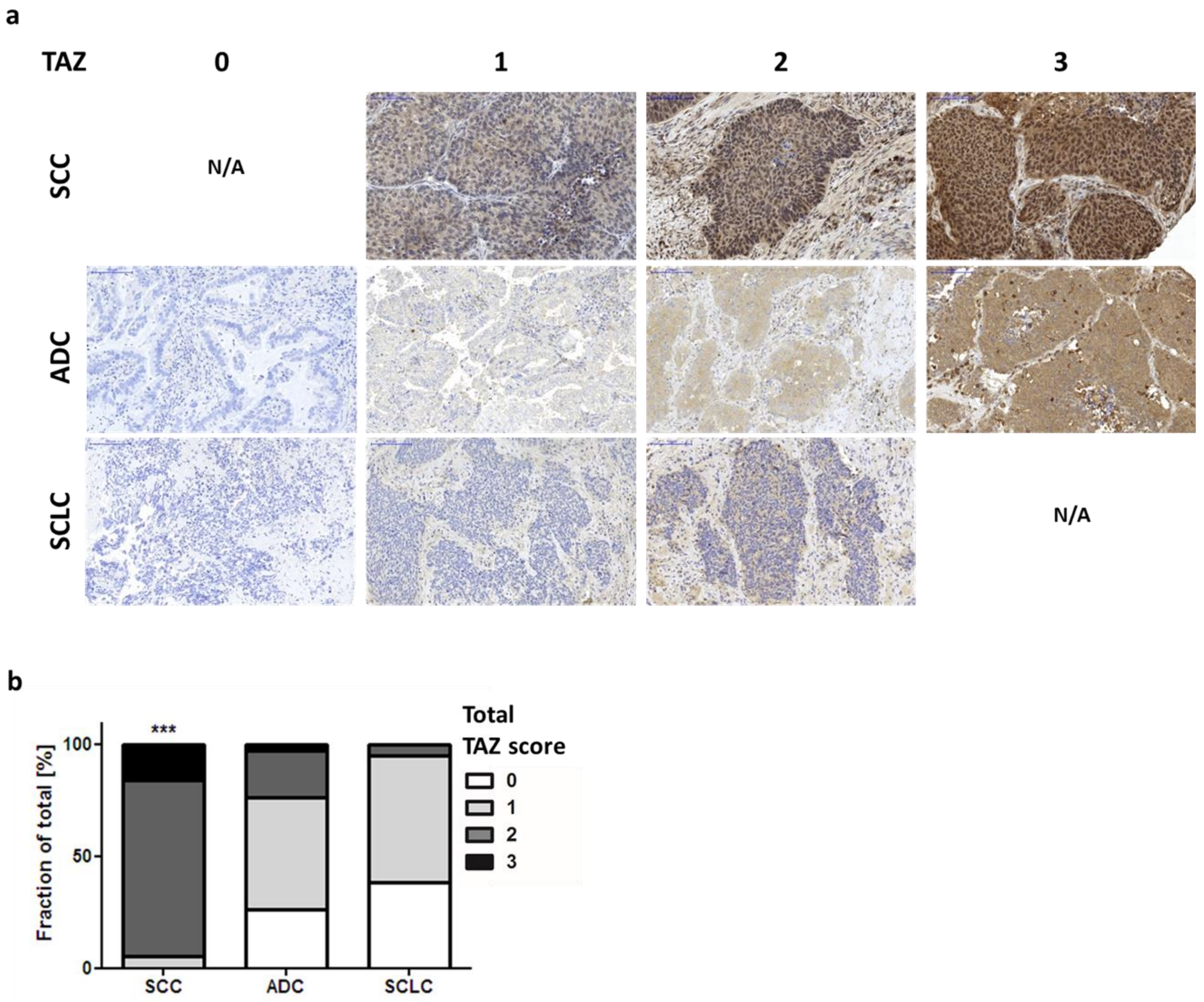

Supplementary Figure 4. TAZ protein expression is associated to SCC. Tissue micro arrays comprising SCC (n=38), ADC (n=37) and SCLC (n=32) were stained for TAZ expression by IHC. (a) Microscopic view at 20x magnification. Bars indicate 100 µm. N/A – not available. (b) TAZ stained sections were scanned, analyzed using the Pannoramic Viewer and scored in terms of total TAZ expression with 0 (no expression), 1 (weak expression), 2 (moderate expression), 3 (strong expression). Statistical analysis was performed using Pearson Correlation (ns – not significant; * p <0.05; ** p <0.01, *** p <0.001).

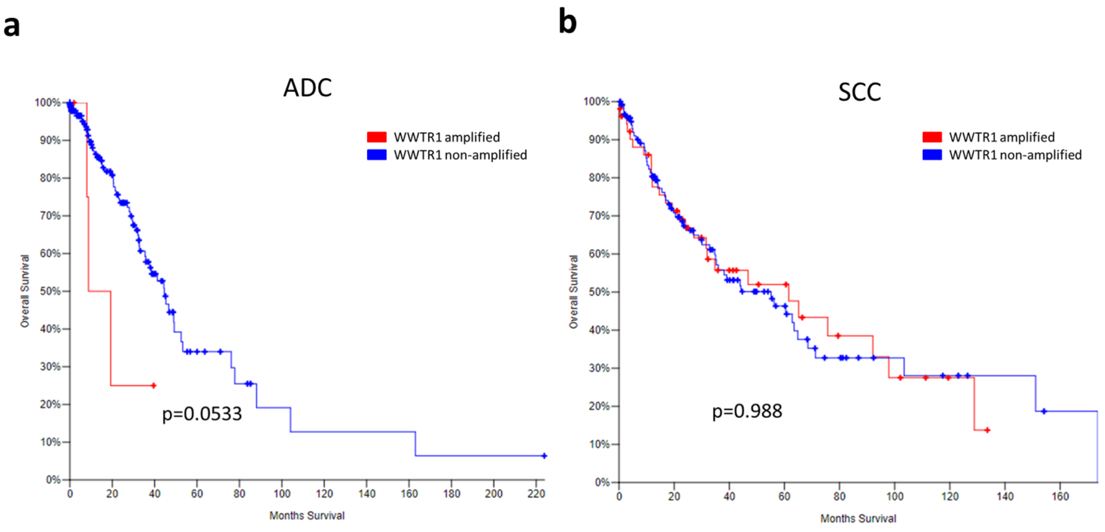

Supplementary Figure 5. WWTR1 amplification is a negative prognostic factor for survival in lung ADCs. Publicly available TCGA datasets providing 230 cases of lung ADCs [22] and 178 cases of lung SCCs [23] are divided in WWTR1 (TAZ) amplified (red) and non-amplified (blue) cases and analyzed concerning overall survival. Statistical analysis was performed using Logrank-Test. P-values are indicated.
